# An Indirect Multimodal Image Registration and Completion Method Guided by Image Synthesis

**DOI:** 10.1155/2020/2684851

**Published:** 2020-06-30

**Authors:** Huan Yang, Pengjiang Qian, Chao Fan

**Affiliations:** ^1^School of Artificial Intelligence and Computer Science, Jiangnan University, Wuxi 214122, China; ^2^Jiangsu Key Lab of Media Design and Software Technology, Jiangnan University, Wuxi 214122, China

## Abstract

Multimodal registration is a challenging task due to the significant variations exhibited from images of different modalities. CT and MRI are two of the most commonly used medical images in clinical diagnosis, since MRI with multicontrast images, together with CT, can provide complementary auxiliary information. The deformable image registration between MRI and CT is essential to analyze the relationships among different modality images. Here, we proposed an indirect multimodal image registration method, i.e., sCT-guided multimodal image registration and problematic image completion method. In addition, we also designed a deep learning-based generative network, Conditional Auto-Encoder Generative Adversarial Network, called CAE-GAN, combining the idea of VAE and GAN under a conditional process to tackle the problem of synthetic CT (sCT) synthesis. Our main contributions in this work can be summarized into three aspects: (1) We designed a new generative network called CAE-GAN, which incorporates the advantages of two popular image synthesis methods, i.e., VAE and GAN, and produced high-quality synthetic images with limited training data. (2) We utilized the sCT generated from multicontrast MRI as an intermediary to transform multimodal MRI-CT registration into monomodal sCT-CT registration, which greatly reduces the registration difficulty. (3) Using normal CT as guidance and reference, we repaired the abnormal MRI while registering the MRI to the normal CT.

## 1. Introduction

Deformable image registration (DIR) is to find the spatial relationship between two or more images and is abundantly used in medical image analysis, such as image fusion, lesion detection, disease diagnosis, surgical planning, and navigation. It is necessary to analyze the relationships among images that were acquired from different viewpoints, at different times, or using different sensors/modalities [1]. Computer Tomography (CT) and Magnetic Resonance Imaging (MRI) are two of the most commonly used medical images in the clinical diagnosis due to the complementary information and multicontrast images they provided. Among them, CT shows precise skeletal location information and electron density information, which is often used for the dose planning of cancer patients. On the other hand, MRI has clear anatomical structures and multiple imaging modalities that enable the detection and segmentation of diseased organs and tissues. The DIR of MRI and CT is essential and a challenging task, due to the inherent structural differences among different modalities and the missing dense ground truth.

Typically, image registration is an iterative optimization process. It requires both a metric that quantifies the similarity between a moving image and a fixed image and an optimization algorithm that iteratively updates the transformation parameters such that the similarity between the images is maximized. The main difficulty of MRI-CT DIR is the definition of the image similarity measure, which is an inherent problem in multimodal image registration. What is more, the registration of MRI and CT is more difficult to perform due to MR images that may be contaminated or damaged by abnormal magnetic fields during acquisition, resulting in unknown deformation. This is a challenging task, not only for image registration but also for image completion.

The significant variations exhibited from images of different modalities make multimodal registration much more troublesome than monomodal registration. Various methods have been proposed to solve multimodal registration tasks, such as mutual information-based [2, 3], elastodynamics-based [4], and learning-based [5, 6]. However, most of these methods are task-sensitive, high-dimensional iterative optimization methods, which are disadvantageous in terms of computational complexity and are difficult to apply in the clinic [7]. Inspired by image fusion, we explore to utilize synthetic images to address the challenge of multimodal image registration and ultimately repair the problematic images. That is, we try to adopt image synthesis algorithms to generate synthetic CT (sCT) from corresponding multicontrast MRI and then use sCT and corresponding CT scans from the same subject to perform image registration. The main idea of our method is to replace the traditional input for multimodal registration with synthetic and real CT images. In this way, multimodal registration is approximately converted into monomodal registration, so MRI-CT registration is indirectly transformed into sCT-CT registration. Guided by the real CT, sCT is generated by the fusion of multiple modality MRI from the same subject, so it has the ability to incorporate the deep features of MRI and the surface features of CT to the greatest extent.

Concerning image synthesis, there are already several existing methods, such as atlas-based [8] and learning-based [9–11]. Atlas-based methods are aimed at generating an atlas among a set of images and then applying the atlas to a new subject. Since this method only relies on geometric transformations, effectiveness and stability are difficult to guarantee. Learning-based methods are achieved by learning a potential nonlinear mapping between source and target domain images. Among them, three themes are mainly used: Variational Auto-Encoder (VAE) [12], Generative Adversarial Network (GAN) [13], and Autoregression [14]. VAE pairs a differentiable encoder network with a decoder network to reconstruct images. However, due to the lack of imperfect similarity measurements, its outputs are often blurry. GAN automatically learns the measurement via a discriminative network and thereby promoting the generative network to generate near-real synthetic images. Inspired by conditional GAN (CGAN) [15] and VAE/GAN [16], we proposed a new deep generative network that combines the idea of VAE and GAN under a conditional process to tackle the problem of sCT synthesis.

The main target of our method is to convert multimodal registration into monomodal registration through a robust image synthesis algorithm and ultimately repair the problematic images. Therefore, our main efforts can be summarized into three aspects:

1. We combined the current popular image synthesis algorithms, i.e., VAE and GAN, to propose a new Conditional Auto-Encoder Generative Adversarial Network, called CAE-GAN. Compared with existing methods, the synthetic images generated by our network are of higher quality and more generalized
2. We utilized the generated sCT to transform multimodal MRI-CT registration into monomodal sCT-CT registration, which greatly reduces the registration difficulty caused by inherent structural differences among different modalities
3. Using normal CT as reference and guidance, through the deformation fields obtained by sCT and CT, we succeed not only in registering multiple modality MRI but also in repairing abnormal MRI

The following parts of the manuscript are arranged as such. Related work, e.g., image synthesis and image registration algorithms, is reviewed briefly in Section 2. The proposed method, CAE-GAN and sCT-guided image registration and completion, is introduced specifically in Section 3. The experimental studies, results, and discussion are presented in Sections 4. Conclusions are given in Section 5.

## 2. Related Work

### 2.1. Image Synthesis

Most traditional image synthesis algorithms include feature extraction, modeling, and target reconstruction, with an assumption that data have a simple formation. They have difficulty in modeling complex patterns of high dimensional, irregular distributions. There have been many recent developments of deep learning-based generative models [17–20], since deep hierarchical architectures are capable of capturing underlying complex features in data. VAE [12] and GAN [13] are two of the most commonly used methods for image synthesis.

VAE is composed of a recognition model and a generative model. Recognition model is also referred to as a probabilistic encoder (E), since given a datapoint *x*, it can produce a distribution (e.g., a Gaussian). Through this distribution, a latent code *z* can be sampled, so that datapoint *x* can be reconstructed from *z* by the generative model (G, also called probabilistic decoder). However, a disadvantage of VAE is that, due to the injected noise and imperfect elementwise measurements such as the squared error, the generated samples are often blurry.

GAN is a generative model that generally includes two subnetworks, a generator G and a discriminator D. G learns a mapping to generate fake samples from random noise, and D tries to discriminate whether the samples are from real or fake. The two networks are like the two sides of a game, and the performance of the two networks gradually improves in the confrontation until D cannot discriminate whether the sample is real or fake. No prior knowledge is needed, and the fake samples are fitted by random noise. This is both an advantage and a disadvantage of original GAN, because the mapping without premodel is too free and broad, it is difficult to get good results. With the proposal of CGAN [15], it adds constraints (condition) to the original GAN to guide image synthesis, which alleviates this problem to some extent. However, the problem still exists, and GAN-based models are hard to converge in the training stage.

There have been some methods which tried to combine GAN and VAE, such as VAE/GAN [16], adversarial autoencoders [21], CVAE-GAN [22]. Inspired by these works, we proposed a new condition-driven deep generative network combining the idea of VAE and GAN, called CAE-GAN. In addition, inspired by the classifier used in Auxiliary Classifier GAN (ACGAN) [23], we reuse the discriminative network as a classifier to further enhance the performance of the network. The detailed network framework is described in Section 3.1.

### 2.2. Image Registration

Generally speaking, image registration is an iterative optimization process, which is achieved by maximizing a predefined image similarity metric calculated from the moving image and the fixed image through an optimization algorithm. Several manually crafted metrics are frequently used, such as the sum of squared differences (SSD), cross-correlation (CC) [24], mutual information (MI) [25], normalized cross-correlation (NCC), and normalized mutual information (NMI). The optimization algorithms are mostly intensity-based [26, 27] and feature-based [28–30]. Actually, image registration generally includes linear (rigid) registration and deformable (nonrigid) registration, where linear registration intends to globally align the two images, and deformable registration is used to correct local deformations. Deformable registration is used to compute a displacement vector for each voxel of an image according to a metric, enabling the estimation of the spatial variations of the anatomy. The displacement vectors are computed to point to the best corresponding location of the voxels in another image according to the metric which is a measure of the image matching.

Among the existing methods, nonparametric deformable registration algorithms are widely used to estimate tissue deformations in highly deformable anatomies due to the advantage of being fast and easy-to-use, such as Demons [31] and Morphons [32]. Demons is an intensity-based registration algorithm, which requires no particular preprocessing nor patient-specific modeling. It is aimed at calculating a regular displacement field which produces a good matching of the intensities in fixed and moving images, along with a measure of the field regularity. However, intensity-based methods are not suitable for registering images with different contrast enhancements. Morphons is a method similar to Demons but phase-based, whose principle is to match transitions rather than intensities, by looking locally at the spatial oscillations in intensities. This method uses Gaussian smoothing as a regularization of the displacement field and additive accumulation during the iterative process. What is more, diffeomorphic transforms [33], which preserve topology and invertibility on the transformation, have shown remarkable superiority in various computational anatomy studies [34–38]. Actually, there have been many learning-based registration methods, such as Quicksilver [39], VoxelMorph [40], and BIRNet [41]. They utilize Convolutional Neural Network (CNN) and spatial transformation function [42] to estimate the similarity measure of the two images or directly predict the transformation parameters of the deformation field. But they are beyond the scope of this article, so we do not introduce much here. In this work, we adopted a registration method based on local phase differences and diffeomorphic accumulation like Diffeomorphic Morphons [38]. The detailed algorithm is presented in Section 3.2.

## 3. Our Proposed Method

Our proposed method consists of two main phases. The first phase is sCT synthesis, two advanced deep learning-based methods, i.e., VAE and GAN, are applied. The second phase is sCT-guided multimodal image registration and image completion. Further details will be given below.

### 3.1. Phase 1: sCT Synthesis Based on Deep Generative Network

**Figure 1:**
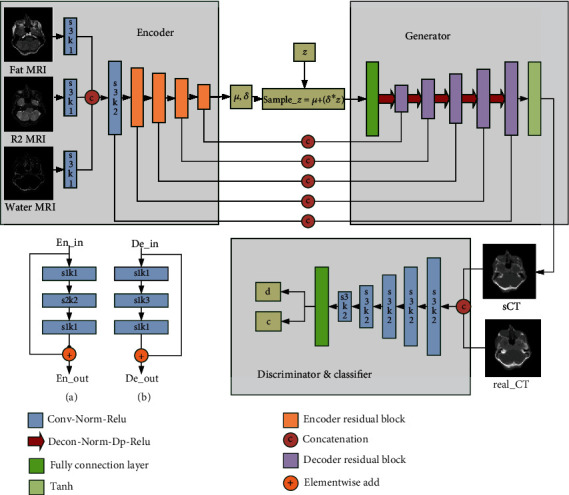
The overall framework of our proposed ACE-GAN. It consists of four subnetworks, encoder (E), generator (G), discriminator (D), and classifier (C). The detailed structure of the encoder and decoder residual block is shown in (a) and (b), respectively. Specifically, s1k1 represents that the convolution stride is 1 and the kernel size is 1 × 1 × 1.

In order to obtain high-quality sCT, we combined the most popular image synthesis methods, i.e., VAE and GAN, to propose a new deep generative network, called CAE-GAN. As shown in Figure 1, it consists of four subnetworks: (1) the encoder network (E), (2) the generator network (G), (3) the discriminator network (D), and (4) the classifier network (C).

The function of networks E and G is the same as that in VAE [12]. The network E maps the input data *x* (e.g., brain fat, R2, and water MR images) to a latent distribution *P*_*z*_ with mean *μ* and standard deviation *δ*. The latent variable sample¯*z* is sampled from *P*_*z*_ through sample¯*z* = *μ* + (*δ*∗*z*) as the input data of G, where *z* is random noise. The function of network G and D is the same as that in GAN [13]. The network G tries to generate synthetic images from the latent variable sample¯*z*, and D tries to discriminate whether the input images are synthetic or real and outputs a probability distribution *d* over input images. They automatically update gradients through adversarial training to improve network performance. The network C is realized by reusing the network structure of D, which only need to change the last activation function of D from sigmoid to softmax. Because the anatomical structure of the brain is relatively simple and clear, its internal tissues can be roughly divided into three types: soft tissue, air, and bone. Therefore, C performs 3-class classification and outputs a probability distribution *c* over the class labels.

However, it is not sufficient to reconstruct images only through the data distribution mapped by E, which results in blurred images. We redesigned the network E and G in the form of concatenation and residual blocks, to form a symmetric encoder-decoder structure. Using this structure, the features of the same resolution at different stages can be fused on the channel via concatenation operation. What is more, we designed encoder and decoder residual blocks to retain more latent features and accelerate network convergence. The use of residual blocks can also avoid possible problems of gradient disappearance or gradient explosion to a certain extent. The detailed structures can be seen in Figure 1.

As for the specific network structure, all convolutional layers (Conv) are followed by normalization (Norm) and the Rectified Linear Unit (Relu) to form a module of Conv-Norm-Relu and all deconvolutional layers (Deconv) are followed by Norm, Dropout (Dp), and Relu to form a module of Deconv-Norm-Dp-Relu. The normalization methods commonly used in deep learning are Batch Normalization (BN) and Instance Normalization (IN). We use IN in our network, because it is normalized from image pixels that are more suitable for image translation tasks. There are three contrast (modality) brain MRI (fat, water, and R2) as input data; we start with three convolutional layers for each input data separately (late-fusion) instead of stacking them as channels (early-fusion). We found late-fuse is better than early-fuse during the experiment.

Using an appropriate and effective loss function is essential for network parameter optimization and performance improvement. As illustrated in Equation (1), we utilize the adversarial loss *L*_adv_ to ensure the adversarial training of G and D. Specifically, G tries to minimize the adversarial loss and D tries to maximize it. In addition, we also introduce an image reconstruction loss *L*_*l*1_ to minimize the difference between synthetic images and reference images, as presented in Equation (2). Therefore, the ultimate objective function is summarized as Equation (3). In these equations below, *x* represents the source domain images (MR), *y* represents the target domain images (CT), and *c* represents class labels. We use a parameter *λ* to control the relative weight of the image reconstruction loss function, set to 100 by default.

(1) $$\begin{array}{c} L_{adv}=E_{y}\left[\log D\left(y\right)\right]+E_{x}\left[1-\log D\left(G\left(E\left(x\right)\right)\right)\right]+E_{y}\left[\log D\left(c\left|y\right.\right)\right]+E_{x}\left[1-\log\left(D\left(G\left(E\left(c\left|x\right.\right)\right)\right)\right)\right] \end{array}$$

(2) $$\begin{array}{c} L_{l1}=E_{x,y}\left[{\left\|y-G\left(E\left(x\right)\right)\right\|}_{1}\right], \end{array}$$

(3) $$\begin{array}{c} L_{\text{total}}=L_{\text{adv}}+\lambda L_{l1}. \end{array}$$

### 3.2. Phase 2: sCT-Guided Multimodal Image Registration and Image Completion

**Figure 2:**
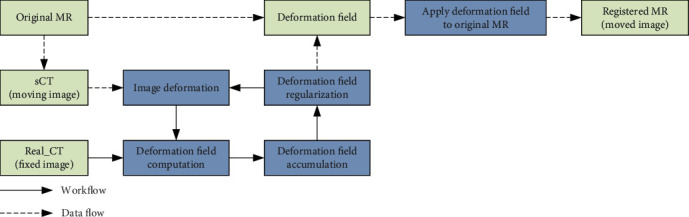
The pipeline of sCT-guided multimodal image registration used in our method.

In our method, we adopted a registration algorithm similar to Diffeomorphic Morphons [38]. This is a nonparametric deformable registration method based on local phase differences at multiple scales. The deformation field estimation is stabilized by using a simple smoothing and downsampling procedure to decompose the fixed and the moving images on several scales. This registration algorithm contains an iterative loop that mainly consists of four interconnected steps, i.e., deformation field computation, deformation field accumulation, deformation field regularization, and image deformation. The idea is to progressively build a proper displacement field by iteratively improving the matching between the fixed and moving images warped by the displacement field, according to a certain metric. The overall framework of this phase is shown in Figure 2.

(1) Deformation field computation: the deformation field is estimated by the dephasing between the local phase of the fixed and moving images. This local phase can be probed at a certain frequency and in a particular direction using quadrature filters [43]. Given the fixed image *f* and moving image *m*, the deformation field *D*_*u*_ can be calculated by solving a weighted least square optimization problem. Accordingly, the global certainty mapping of deformation field can be expressed mathematically as
  (4) $$\begin{array}{c} \Theta_{c}\left(f,m\right)=\underset{k}{\sum }c_{k}\left(x\right), \end{array}$$
  where *c*_*k*_(*x*) = *A*_*f*_(*x*; *k*)*A*_*m*_(*x*; *k*) is the certainty mapping of the filter mentioned above, with *A*_*f*_(*x*; *k*) and *A*_*m*_(*x*; *k*) denoting the amplitudes of the fixed and moving images, respectively. Then, this update of certainty map will be combined with an accumulated certainty computed from previous iterations
(2) Deformation field accumulation: the complete accumulated field is computed as a weighted sum of the update field and the previous accumulated field. The weights are given by the certainty on the update field and the accumulated certainty map. Besides, some new tricks are adopted in this method. Let the deformation field be represented by a vector field *D*, and let $\Delta \overset{\Delta }{=}Id+D$ denote the deformation operation, where Id denotes the identity deformation: $\text{Id}\left(x\right)\overset{\Delta }{=}x$. Then, the accumulation process can be expressed as
  (5) $$\begin{array}{c} D_{1}⊕D_{2}\overset{\Delta }{=}\Delta_{1}\circ \Delta_{2}-\text{Id}, \end{array}$$
  where ∘ represents the common function composition operation, denoting the warping of two objects. The composition will remain diffeomorphic in the case of two diffeomorphic deformation fields
(3) Deformation field regularization: field regularization is in order to get a smoother transformation and reduce the impact of image noise on the registration output. In our method, regularization is achieved by using a normalized convolution [44] of the deformation field by the Gaussian kernel. In addition, the certainty map obtained from local phase computation is also used to adjust the importance of the target locations, i.e., the higher significance is attached to locations with larger values in certainty map
(4) Image deformation: the three steps mentioned above are performed iteratively a certain number of times at each scale from coarse to refine, until it reaches a certain stopping criterion. Then, the resulting regularized deformation field is used to warp the moving image to obtain the registered image

In our method, we use sCT (moving image) and real CT (fixed image) as input and then apply the optimal deformation field output to original MRI, so as to obtain the final registered MRI (moved image). Because sCT is completely generated on the basis of MRI, which is representative of the characteristics of the original MRI, so the deformation field obtained by registering sCT and real CT can be used to register original MRI to reference normal CT. In this process, we transformed multimodal MRI-CT registration into monomodal sCT-CT registration and repaired the problematic MRI at the same time. Thus, our overall approach consists of two interrelated phases, i.e., sCT synthesis based on deep generative network and sCT-guided multimodal image registration and image completion. To some extent, the image registration and completion results in the second phase largely depend on the quality of synthetic CT generated in the first phase.

## 4. Experiment and Discussion

### 4.1. Experiment Setup

The dataset used in our experiment includes nine sets of brain MR-CT images from nine healthy subjects after providing informed consent. For each subject, there are three types of MR sequences, e.g., fat, water, and R2, and corresponding CT scans from the same anatomy. To simulate the deformation and other unknown corruption that MR images may encounter during acquisition, we randomly warped the original MR images with salt-and-pepper noise. To make full use of these brain MR-CT images, we adopted a leave-one-out (LOO) strategy to get unbiased cross-validation results of each subject. LOO trains the network on all but one case and tests on that case and then repeats this process on each case. In other words, for the sCT of each subject, we obtained it by testing on the network which was trained with the images from the other nine subjects. To fit the memory and computing resources of the computer, we divided the original MR and CT images with the resolution of 256 × 256 × 256 into many 64 × 64 × 64 patches. The training and testing patches for the network are extracted by sliding over the original images. After the network converges, the generated patches are then recombined to form a complete sCT.

In order to demonstrate the superiority of our proposed framework, two image synthesis methods, i.e., FCN-based deep generative network and FCM-based clustering algorithm, are chosen as opponents for the comparison of sCT synthesis. On the other hand, two classical traditional registration methods, i.e., Diffeomorphic Morphons (D_ Morphons, a method based on local phase differences) [38] and Diffeomorphic Demons (D_Demons, a method based on the sum of squared differences) [37], are selected for the comparison of image registration. In all non-learning-based approaches, the methods using diffeomorphic transforms can preserve topology and the invertibility of the transformation, so they are still effective and representative methods nowadays. We utilize the OpenREGGUI, an open-source image registration package to implement these registration methods.

Our experimental studies were carried out on a computer with an Intel Xeon® CPU E5-2640 V4, GeForce RTX 2080 Ti GPU using the Ubuntu 16.04 (64 bit) operating system. We use TensorFlow (Google, California, USA) and MATLAB 2016a (MATHWorks, Natick, MA, USA) to implement our proposed method.

### 4.2. Metrics for Evaluation

To demonstrate the effectiveness of our proposed deep generative network, we use the Mean Absolute Error (MAE) and the Pearson Correlation Coefficient (CC) to quantitatively measure the similarity between generated and reference images. That is, the values (see Table 1) are calculated by measured CT and sCT generated by different algorithms, i.e., FCN-based, FCM-based, and our proposed CAE-GAN. A lower value of MAE and higher value of CC indicate a smaller error and higher quality of generated images.

As for the evaluation of image registration, two metrics are used in our research: the mutual information (MI) and the sum of local phase differences (SLPD). Giving the fixed image *f* and moved image *m*, the MI measures the mutual dependence between two images and can be defined as

(6) $$\begin{array}{c} \text{MI}\left(f;m\right)=\underset{f\in F}{\sum }\underset{m\in M}{\sum }p\left(f,m\right)\log\left(\frac{p\left(f,m\right)}{p\left(f\right)p\left(m\right)}\right), \end{array}$$

where *p*(*f*, *m*) is the joint probability function of *f* and *m*, and *p*(*f*) and *p*(*m*) are the marginal probability distribution functions of *f* and *m*, respectively. The SLPD is calculated as the sum of the local phase differences in all directions between two images. Mathematically, it can be defined as

(7) $$\begin{array}{c} \text{SLPD}=\sum \left(\sin\left(\Delta \varphi \right)\right), \end{array}$$

where Δ*φ* denotes the local phase differences between two images. A lower value of SLPD and higher value of MI indicate higher registration accuracy and better results.

### 4.3. Results and Discussion

**Figure 3:**
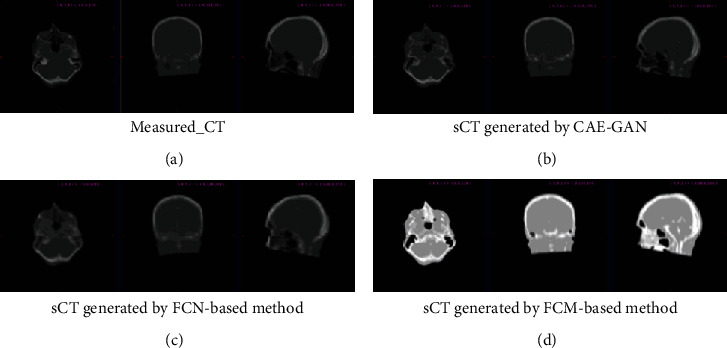
Results of different image synthesis algorithms on subject 1. (a) is the measured CT, and (b)–(d) are the synthetic CT generated by different methods. Each slice image is viewed from three perspectives, transverse, coronal, and sagittal, from left to right. It can be seen that (b) is the closest to the measured CT with high synthesis accuracy and quality.

The sCT synthesis phase is an important component of our proposed sCT-guided multimodal image registration and completion method. We separately ran CAE-GAN, FCN_based, and FCM_based methods on the brain images obtained from 9 subjects. To illustrate the effectiveness of our proposed deep generative network, we calculated the evaluation metrics between measured CT and sCT that are generated by FCM_based, FCN_based, and our CAE-GAN methods, respectively. The values of MAE and CC of different methods for each subject are listed in Table 1. As can be seen from the bold fonts (best) marked in the table, except for sub2, our proposed method has lower MAE and higher CC than other methods, which demonstrates that our method produces more precise and higher quality synthetic images.  Figure 3 shows the results of sCT synthesis by different methods on subject 1. From these synthetic slices, we can clearly see that the sCT generated by our method is very similar to the measured CT with clearer internal tissue textures and higher-quality synthesis effects. In addition, we can conclude that deep learning-based methods (FCN_based and our method) are superior to traditional machine learning-based methods (FCM_based), which can be seen from Table 1 and Figure 3.

**Figure 4:**
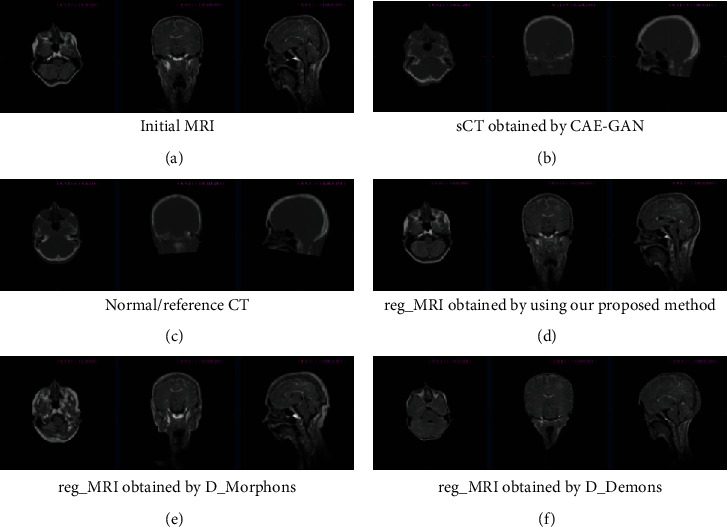
Results of image registration and completion on subject 1. (a) is the initial contaminated MRI, (b) is the synthetic CT as an intermediary, (c) is the normal CT as guidance and reference, and (d)–(f) are registered MRI obtained by using different registration methods. Each slice image is viewed from three perspectives, transverse, coronal, and sagittal, from left to right. The results demonstrate that the reg_MRI obtained by our method has a high matching of tissue deformation and spatial location with the reference CT and repairs the abnormal MRI.

For the purpose of the performance comparison of different registration methods, we measured the MI and SLPD introduced above for each subject and listed in Table 2. They are calculated by measured CT and registered MRI obtained from different registration methods. It can be seen from evaluation metrics that our proposed sCT-guided image registration method has similar and even more superior performance to other classical traditional registration methods. From Figure 4 that shows the results of registration and completion on subject 1, we can clearly see that the registered MRI obtained using our method has a high matching of tissue deformation and spatial location with the corresponding reference CT and it repairs the initial abnormal MRI. Besides, it can be illustrated that the matching of structures in images based on intensity is unsuitable for registering variable contrast images, as can be seen from Figure 4(f). It may lead to uncontrollable abnormal tissue deformation, making the registration results not credible. What is more, considering that in clinical applications, time is very important. We recorded the total time consumption used in our experiments, which is listed in Table 3. Our proposed method in this work consists of two interrelated phases, i.e., sCT synthesis and image registration. Once the image synthesis network is trained well, the total time cost of our method is less than two minutes, which is much more efficient than the other two registration methods. Thus, our method obviously consumes less time and obtains better results than either of these methods.

Overall, in our method, we utilized a deep generative network to obtain sCT and then used sCT as an intermediary to convert the problem of multimodal MRI-CT registration into the problem of monomodal sCT-CT registration. In this way, we successfully addressed the challenges of multimodal registration caused by the significant differences between different contrast images and reduced the difficulty of registration. We utilized a traditional deformable registration method based on local phase differences and diffeomorphic accumulation to perform the image registration process and enable the completion of problematic MR images. We did not use the latest popular deep learning-based registration algorithms due to insufficient data and the lack of optimal optimization goals. The deep generative models are relatively mature now, but the deep registration methods still face many challenges, especially for multimodal registration. It is very difficult to design a common optimal similarity index to measure the accuracy of registration. Due to the inherent defects of deep learning, strong data dependence, and poor interpretability, model stability and generalization are limited in practice. Besides, one disadvantage of our method is that we require normal CT scans from the same subject to serve as a guide and reference to enable indirect multimodal registration and completion, which may have trouble in practical application scenarios. Nevertheless, with limited data, we have proved the effectiveness of our proposed deep generative network and indirect multimodal image registration and completion method. Compared with the existing methods of multimodal registration, our method exhibits clear superiority.

## 5. Conclusion

In this paper, inspired by image synthesis, we utilized synthetic CT as an intermediary to solve the challenging multimodal image registration problem. We proposed a sCT-guided multimodal MRI registration and completion method and designed a new deep generative network called Conditional Auto-Encoder Generative Adversarial Network (CAE-GAN), which combined the idea of VAE and GAN, to obtain high-quality synthetic CT. We conducted experiments on brain MR-CT images provided by nine subjects. The experimental results illustrated that our designed deep generative network can yield high-quality synthetic images and our proposed image registration method can exhibit clear superiority in registration accuracy and time consumption compared with other methods.

## Figures and Tables

**Table 1:** Performance comparison of three image synthesis algorithms. The best results of each subject are presented in bold.

Sub	MAE			CC		
	FCM_based	FCN_based	CAE-GAN	FCM_based	FCN_based	CAE-GAN
1	157.81	77.11	**58.88**	0.75	0.94	**0.96**
2	176.91	**95.24**	98.44	0.61	**0.91**	0.89
3	157.94	99.77	**69.99**	0.71	0.90	**0.92**
4	178.49	87.34	**67.49**	0.74	0.92	**0.93**
5	165.03	103.34	**99.32**	0.70	0.89	**0.89**
6	152.67	105.57	**97.54**	0.71	0.89	**0.89**
7	169.38	82.08	**78.96**	0.67	0.92	**0.93**
8	146.57	94.91	**71.76**	0.67	0.89	**0.92**
9	171.82	110.18	**88.94**	0.79	0.88	**0.90**

**Table 2:** Performance comparison between our proposed method and other registration methods. The best results are presented in bold.

Sub	MI			SLPD		
	Ours	D_Morphons	D_Demons	Ours	D_Morphons	D_Demons
1	**0.9482**	0.9465	0.9466	**1.6159*E* + 05**	1.6803*E* + 05	1.7738*E* + 05
2	**0.9220**	0.9177	0.9161	**2.6173*E* + 04**	4.1678*E* + 04	2.9328*E* + 04
3	**0.9338**	0.9307	0.9328	**1.9668*E* + 04**	1.1733*E* + 05	5.8928*E* + 04
4	**0.9397**	0.9340	0.9386	**6.0200*E* + 04**	7.6741*E* + 04	9.0167*E* + 04
5	**0.9251**	0.9184	0.9238	**1.0450*E* + 05**	1.3337*E* + 05	1.5216*E* + 05
6	0.9316	**0.9456**	0.9311	9.4646*E* + 04	**8.2707*E* + 04**	9.2987*E* + 04
7	**0.9437**	0.9378	0.9435	**1.1808*E* + 05**	1.3797*E* + 05	1.2557*E* + 05
8	**0.9414**	0.9348	0.9411	**1.4943*E* + 05**	1.6041*E* + 05	1.8494*E* + 05
9	**0.9286**	0.9271	0.9278	**4.3636*E* + 04**	1.6514*E* + 05	7.6691*E* + 04

**Table 3:** Time consumption of three image registration methods.

Sub	Ours			D_Morphons	D_Demons
	sCT synthesis (s)	Registration (s)	Total time (s)	Total time (s)	Total time (s)
1	3.5	84	87.5	450	376
2	3.5	84	87.5	582	380
3	3.5	89	92.5	583	353
4	3.7	90	93.7	586	354
5	3.5	85	88.5	585	355
6	3.6	84	87.6	585	356
7	3.5	82	85.5	582	353
8	3.5	89	92.5	582	352
9	3.6	92	95.6	583	378

## Data Availability

The data used to support the findings of this study are available from the corresponding author upon request.
